# A quantitative atlas of histone modification signatures from human cancer cells

**DOI:** 10.1186/1756-8935-6-20

**Published:** 2013-07-05

**Authors:** Gary LeRoy, Peter A DiMaggio, Eric Y Chan, Barry M Zee, M Andres Blanco, Barbara Bryant, Ian Z Flaniken, Sherry Liu, Yibin Kang, Patrick Trojer, Benjamin A Garcia

**Affiliations:** 1 Department of Molecular Biology, Princeton University, Princeton, NJ 08544, USA; 2 Department of Chemical Engineering, Imperial College London, London SW7 2AZ, UK; 3 Constellation Pharmaceuticals, Inc., Cambridge, MA 02142, USA; 4 Epigenetics Program, Perelman School of Medicine, University of Pennsylvania, Smilow Center for Translational Research, 3400 Civic Center Blvd., Bldg 421, Philadelphia, PA 19104, USA; 5 Department of Biochemistry and Biophysics, Perelman School of Medicine, University of Pennsylvania, Smilow Center for Translational Research, 3400 Civic Center Blvd., Bldg 421, Philadelphia PA 19104, USA

## Abstract

**Background:**

An integral component of cancer biology is the understanding of molecular properties uniquely distinguishing one cancer type from another. One class of such properties is histone post-translational modifications (PTMs). Many histone PTMs are linked to the same diverse nuclear functions implicated in cancer development, including transcriptional activation and epigenetic regulation, which are often indirectly assayed with standard genomic technologies. Thus, there is a need for a comprehensive and quantitative profiling of cancer lines focused on their chromatin modification states.

**Results:**

To complement genomic expression profiles of cancer lines, we report the proteomic classification of 24 different lines, the majority of which are cancer cells, by quantifying the abundances of a large panel of single and combinatorial histone H3 and H4 PTMs, and histone variants. Concurrent to the proteomic analysis, we performed transcriptomic analysis on histone modifying enzyme abundances as a proxy for quantifying their activity levels. While the transcriptomic and proteomic results were generally consistent in terms of predicting histone PTM abundance from enzyme abundances, several PTMs were regulated independently of the modifying enzyme expression. In addition, combinatorial PTMs containing H3K27 methylation were especially enriched in breast cell lines. Knockdown of the predominant H3K27 methyltransferase, enhancer of zeste 2 (EZH2), in a mouse mammary xenograft model significantly reduced tumor burden in these animals and demonstrated the predictive utility of proteomic techniques.

**Conclusions:**

Our proteomic and genomic characterizations of the histone modification states provide a resource for future investigations of the epigenetic and non-epigenetic determinants for classifying and analyzing cancer cells.

## Background

The search for the molecular properties that define cancer has provided important insights into the general cancerous state, as exemplified by the characterizations of commonly mutated tumor suppressors [1]. Yet cancer is not a single disease. Thus, it is not sufficient to only explore what broadly distinguishes the cancerous state from the otherwise healthy state. It is essential to identify in addition the molecular properties that distinguish a given class of cancer cells from another. By understanding both the general and specific properties defining cancer, one could design a comprehensive classification of cancer cells rooted in their underlying molecular biology and provide a resource useful for diagnosis and treatment.

Many of the properties that characterize cancer phenotypes operate at the genetic level, namely transcriptional states and gene mutations. Genomic approaches that collate gene expression patterns and catalog DNA sequence information have served as a vital platform for the profiling of a variety of cancer cells [2]. Absent from most genomic-based classifications of cancer is epigenetic information, which is a grasp of the physical *in vivo* state of genes. In eukaryotic cells, DNA typically exists in a complex with histones known as chromatin. Chromatin can be generalized as an array of tandem core nucleosomes individually composed of two copies each of histones H2A, H2B, H3 and H4 coiling approximately 147 bp of DNA. The consequences of DNA existing in a complex with histones are enormous. First, electrostatic interactions between every 10 bp of DNA and the histone octamer occlude various non-histone proteins from binding those nucleotides. Second, the stability of the nucleosome results in the need for ATP-dependent chromatin remodelers for the positioning and alteration in nucleosome structure. Because of these associations between histones and DNA, it is clear that many nuclear events, such as transcription, DNA damage repair and replication, are impacted by the nucleosome. It is also clear that these events broadly contribute to cancerous development when mis-regulated, for instance, the failure to detect or repair double stranded DNA breaks. Thus, an understanding of the cancerous state requires an understanding of the chromatin state.

Many correlations between DNA-dependent events and histones also occur at the level of histone post-translational modifications (PTMs), namely lysine acetylation (ac), mono- (me1), di- (me2), and trimethylation (me3). Most histone PTMs do not alter nucleosome structure, rather the majority of PTMs are thought to recruit non-histone proteins via specialized PTM binding domains, such as the bromo, chromo and PHD domains. The binding of these non-histone proteins to chromatin can mediate many of the aforementioned events in a paradigm known as the histone code hypothesis [3]. For instance, trimethylated lysine 27 of histone H3 (H3K27me3) is recognized by Polycomb group (PcG) proteins, which maintain epigenetic silencing. Establishing this paradigm is the enzymatic regulation of histone modification states, such as histone acetyltransferases (HATs) and methyltransferases (HMTs), which acetylate and methylate histones respectively, and histone deacetylases (HDACs) and demethylases (HDMs), which convert the acetylated and methylated states, respectively, to the unmodified state. For example, H3K27me3 is formed by the EZH2 methyltransferase [4].

Between histone-modifying enzyme activities that regulate histone PTM states and histone-binding proteins that effect DNA-dependent processes, cancer development has been linked to histone modifications at various levels [5]. One example linking cancer with a histone PTM-binding protein includes associations between bromodomain-containing BRD3 and BRD4 proteins, which recognizes histones H4 acetylated at K5, K8 and K12 [6], and PAFc and SEC elongation factors in MLL fusion leukemias [7], as well as translational fusions between BRD4 and NUT proteins in certain squamous carcinomas [8,9]. An example linking cancer with a histone PTM modifying enzyme protein would be the commonly observed mutations in EZH2 HMT in certain lymphomas and myelodysplastic syndromes that reduce the catalytic activity to trimethylate H3K27 [10,11]. What these examples emphasize is that first, despite the pervasive nature of histone PTMs, specific cancer phenotypes arise from alterations in the chromatin PTM landscape and, second, there may potentially be many other histone modifications of equal or greater importance in cancer biology.

Given the relevance of histone PTMs to cancer, heretofore understood primarily using low-throughput and often inferential approaches, there is a need for a comprehensive profiling of cancer cells concentrated specifically on their chromatin modification states. Here we applied mass spectrometry-based proteomic techniques to quantify global abundances of histone PTMs from 24 commonly used cell lines, the majority of which are of cancerous lineages. Additionally, we applied microarray-based genomic techniques to monitor transcript levels of various histone-modifying enzymes. The proteomic and transcriptomic analyses were generally consistent in classifying which lines were similar, based on their chromatin PTM patterns and chromatin modifying enzyme levels, respectively. Among our results, H3K27 methylation was highly increased in breast cancer cells. Moreover, depletion of EZH2 led to decreased tumor formation in a mouse xenograft model, consistent with the known role of EZH2 in the pathogenesis of other cancers. In summary, this study constitutes an “atlas” of histone PTMs across the reported lines, and using this atlas, one may potentially attain a novel molecular classification of cancer cells based on their chromatin modification landscape. We believe this dataset will motivate future studies in the cancer chromatin field.

## Results

### A chromatin atlas of cancer lines quantifying transcript levels of chromatin-modifying enzymes

**Figure 1:**
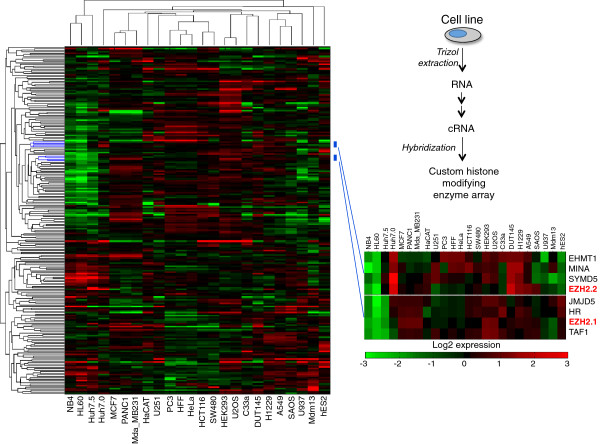
**Gene expression analysis of histone modifying enzymes across cell lines.** Hierarchical clustering of the 24 cell lines along the horizontal axis was performed based on transcript abundances of HATs, HDACs, HMTs and HDMs along the vertical axis, where an orthogonal clustering was similarly performed. The insert magnifies two clusters containing the two transcript variants of EZH2 HMT (EZH2.1 and EZH2.2). Data are shown as log_2_ expression relative to the reference RNA library.

For our chromatin-centric atlas, we examined 24 cell lines from a diverse variety of tissue origins, including cervix, prostate, lung and breast tissues (Table 1). The majority of these lines are cancer cells, and the remaining non-cancer lines include 293, HFF, HaCAT and hESC cells. To explore whether different cell lines would possess different chromatin profiles, we harvested RNA from all the lines and performed gene expression analysis using a custom microarray with multiple probes to 224 human HATs, HDACs, HMTs and HDMs (Figure 1).

**Table 1:** Tissue culture cell lines used in this study

**Cell line**	**Media**	**Comment**
1. 293	DMEM	Embryonic kidney
2. A549	DMEM	Adenocarcinomic alveolar basal epithelial
3. C33a	DMEM	Cervical carcinoma
4. DUT145	DMEM	Prostate cancer
5. H1229	RPMI	Lung carcinoma
6. HaCAT	RPMI	Immortalized, non-tumorigenic keratinocyte
7. HCT116	McCoy 5A	Colorectal carcinoma
8. HeLa	Joklik	Cervical carcinoma
9. hESC2	DMEM	Human embryonic stem cells
10. HFF	DMEM	Human foreskin fibroblasts
11. HL60	IMM	Promyelocytic leukemia cells
13. Huh7.0	DMEM	Hepatocarcinoma
14. Huh7.5	DMEM	Hepatocarcinoma (RIG-I-deficient)
14. MCF7	DMEM	Breast carcinoma
15. MDaMB231	L15	Breast carcinoma
16. Mdm13	DMEM	Monocyte-derived macrophages
17. NB4	RPMI	Acute promyelocytic cell line
18. PANC1	DMEM	Pancreatic carcinoma, epithelial-like
19. PC3	DMEM	Prostate cancer
20. SAOS	McCoy 5A	Epithelial-like osteosarcoma cell line
21. SW480	L15	Colon adenocarcinoma
22. U2OS	DMEM	Osteosarcoma
23. U251	DMEM	Glioblastoma
24. U937	RPMI	Leukemic monocyte lymphoma

Our hypothesis is that differences in the chromatin state can potentially give rise to the distinguishing characteristics of cancer cell lines. Indeed, cell lines that originate from a common tissue type generally cluster together (Figure 1). The two breast lines in our study, MCF7 and MDA-MB231, exhibited similar expression profiles across the histone modifying enzymes (Wilcoxon sign rank, zval = −0.9207, *P* = 0.3572). While it may be expected that two lines from different tissue types have significantly different expression profiles, for instance, between MDA-MB231 and the leukemia HL60 line (Wilcoxon sign rank, zval = −4.2728, *P* = 1.9305 × 10^-5^), some lines from different tissue types have similar expression profiles. For instance, similar profiles were observed between the HEK293 line and the U2OS cell line (Wilcoxon sign rank, zval = −1.7466, *P* = 0.0807) to a modest extent and between the cervical HeLa line and the PC3 prostate line (Wilcoxon sign rank, zval = −1.5303, *P* = 0.1259). What these results suggest is that the expression levels of chromatin modifying enzymes alone are insufficient for distinguishing chromatin modification patterns between cancer cell lines, and that other factors may regulate the activity of these enzymes, such as post-translational modifications of the chromatin modifying enzymes, themselves likely influence the chromatin state [12]. Moreover, these results also suggest that cancers of distinct origins may have similar transcriptomic profiles, which should be helpful when considering treatment options.

### Quantification of histone PTM abundances for a complementary proteomic atlas

The 24 cell lines exhibited a diverse range of gene expression patterns for the various histone modifying enzymes. Although gene expression analysis revealed both expected and unexpected correlations across the lines, a critical limitation of such analyses is the inability to extrapolate enzyme activity from enzyme transcript abundance. It is the activity of the chromatin modifying enzymes that is most informative for designing an accurate classification of cell lines based on their chromatin states. Thus, to complement our transcriptomic analysis, our strategy to extrapolate enzyme activity was to determine the abundances of the enzyme substrates and enzyme products, namely the steady state histone PTM levels.

**Figure 2:**
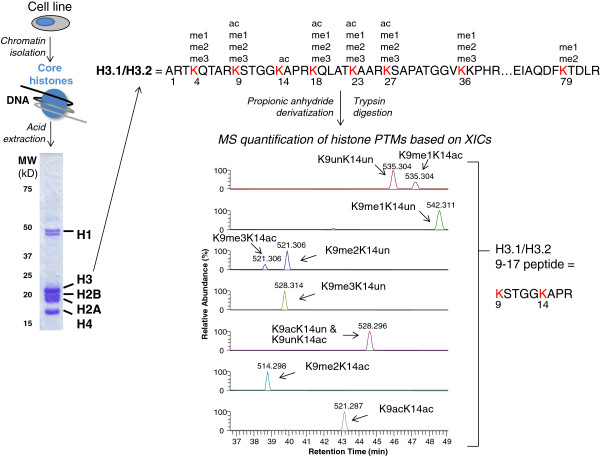
**Proteomic strategy for histone PTM quantification.** Each of the 24 lines was similarly processed to yield the core and linker histones. Histones were derivatized with propionic anhydride to prevent trypsin digestion at the unmodified and monomethylated lysines and, consequently, peptides of uniform length are produced that span the same modified residue (in this example, lysines 9 and 14 of histone H3). Relative quantification is achieved by peak integration of the extracted ion chromatograms of each charge state (the +2 charge state is shown) for each peptide (each colored row, with m/z shown). Note that H3K9acK14un and H3K9unK14ac co-elute in this experiment, thereby requiring tandem MS sequencing to resolve both peptides for separate quantification.

In order to determine the relative abundances of histone modifications for each line, we utilized our Bottom Up MS protocol, which has also been adapted by multiple laboratories [13-15]. Here, we generated ArgC-like fragments from acid-extracted histones and analyzed the peptides on a hybrid Orbitrap-linear ion trap quadrupole mass spectrometer (ThermoFisher Scientific, Carlsbad, CA, USA) (Figure 2). With this approach, we reproducibly obtained greater than 90% sequence coverage for histones H3 and H4 and were able to quantify 37 unique histone modification patterns on histone H3 and 19 patterns on histone H4 across every cell line (Additional file 1). We normalized all detected PTMs or modified forms of a common peptide backbone to each other. For instance, we would normalize the measured abundances of all the observable modified forms of the 9 to 17 histone H3 peptide (KSTGGKAPR), which spans lysine 9 and lysine 14, against each other. The normalization would occur independently of all observed modified forms of another histone peptide, such as the 18 to 26 peptide (KQLATKAAR). Because of our normalization, our PTM values for a given peptide backbone are not necessarily independent of each other. Thus, for *n* modified forms of a given peptide, there are *n* – 1 degrees of freedom. Because we quantified five different histone H3.1/H3.2 peptide backbones: namely the 3 to 8 peptide TKQTAR, the 9 to 17 peptide KSTGGKAPR, the 18 to 26 peptide KQLATKAAR, the 27 to 40 peptide KSAPATGGVKKPHR, and the 73 to 83 peptide EIAQDFKTDLR, we have *n* – 5 degrees of freedom along the histone H3 PTM axis. Here we have supplied the normalized values not only in terms of fold enrichment, but also relative abundance, thus one can gauge the significance of the change based on the magnitude of the PTM abundance. Additionally, lower abundance marks require greater fold change to be deemed significant and, conversely, higher abundance marks require lower fold change to be deemed significant.

### A chromatin atlas of cancer lines focused on relative levels of chromatin modifications

**Figure 3:**
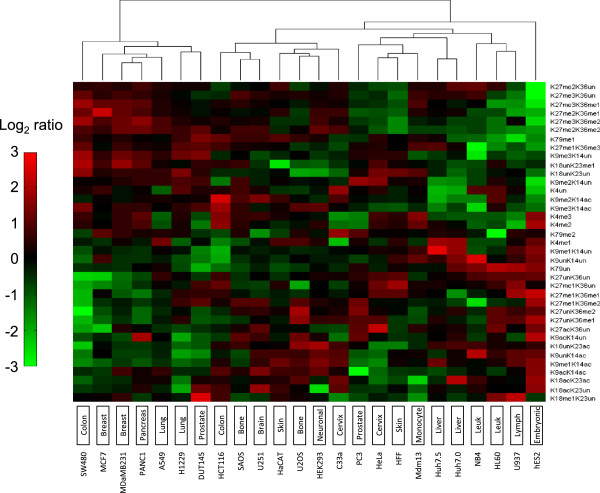
**Proteomic analysis of histone H3 PTMs across cell lines.** Hierarchical clustering of the 24 lines was performed based on abundances of histone H3 PTMs (shown in right vertical axis). Cell line tissue type is shown in the bottom horizontal axis. Data shown as log_2_ enrichment of histone PTM levels for a given line (biological replicates of 3 to 7) based on average PTM levels across all 24 lines.

**Figure 4:**
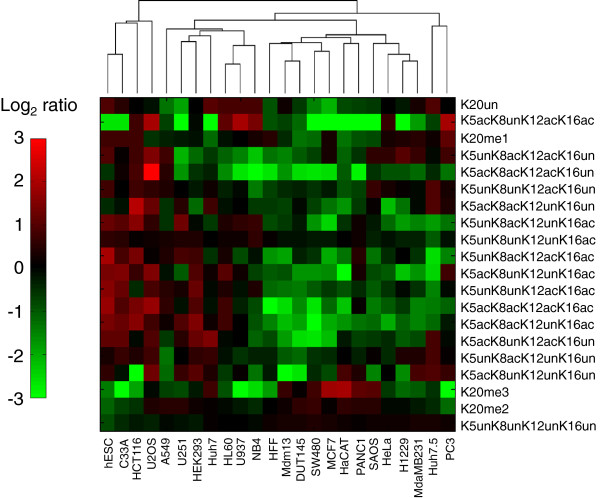
**Proteomic analysis of histone H4 PTMs across cell lines.** Hierarchical clustering of the 24 lines was performed based on abundances of histone H4 PTMs (shown in right vertical axis). Data are shown as log_2_ enrichment of histone PTM levels for a given line based on average PTM levels across all 24 lines.

We report the modification levels on histone H3 (Figure 3) and histone H4 (Figure 4) for each of the 24 cell lines across the average relative abundance of all the lines. For example, we determined the enrichment of the relative abundance of H3K27me3K36un for MCF7 against the average relative abundance of H3K27me3K36un across the 24 lines. The actual and average relative abundances for each line are also provided (Additional file 1). Analogous to the transcriptomic analysis, cell lines of similar tissue origin have similar chromatin modification profiles. For instance, enrichment of a given PTM in the breast line MCF7 is generally accompanied with similar enrichment of the same PTM in the other breast line MDA-MB231 (Figure 3). In particular, the differences in PTM relative abundance between the Huh7.0 and Huh7.5 were comparable to the differences between MCF7 and MDA-MB231 (Wilcoxon sign rank, z = −0.0830, *P* = 0.9339) and between A549 and H1229 (Wilcoxon sign rank, z = −0.3093, *P* = 0.7571). In contrast, the differences in PTM abundances between PANC1 and NB4 was significantly greater than the differences between Huh7.0 and Huh7.5 (Wilcoxon sign rank, *z* = −4.5334, *P* = 5.8034 × 10^-6^), implying that PANC1 and NB4 have different chromatin modification profiles. Yet there were exceptions to the generality that lines of different tissue origins will have different histone PTM patterns. For instance, the differences between SAOS and U251 were comparable to the differences between MCF7 and MDA-MB231 (Wilcoxon sign rank, z = −0.2640, *P* = 0.7918) and Huh7.0 and Huh7.5 (Wilcoxon sign rank, z = −0.5959, *P* = 0.5512), implying a similar chromatin modification profile of the SAOS and U251 lines despite originating from different tissues.

Applying our analytic software, we successfully quantified the 9 to 17 H3 peptides (KSTGGKAPR) containing serine 10 phosphorylation that otherwise would be difficult to quantify manually due to their low abundance. In general, most of the 24 lines had less than 1% total occurrence of H3S10 phosphorylation, with the notable exceptions of Panc1, Huh7.0 and Huh7.5 that contain 4.4, 3.7 and 4.3% relative abundance of phosphorylated peptides, respectively (Additional file 2). Since the cell lines were not synchronized across the cell cycle, we hypothesize that the levels of H3S10 phosphorylation proportionately reflect the percentage of cells in an asynchronous population that were in M phase at the time of harvest. Furthermore, when we compared the modification states of H3K9 and H3K14 depending on the modification state of H3S10, we observed that the H3K9 and H3K14 modification patterns differed based on the phosphorylation state of H3S10. In particular, H3S10 phosphorylation generally co-occurred with H3K9me3K14un, even when this modification is not particularly abundant for a given line (Additional file 2). It is possible that this trend reflects the methyl-phospho switch model [16], which proposes that a key function of H3S10 phosphorylation is to block HP1 from binding to H3K9me2 or H3K9me3. Thus, H3S10ph would be expected to co-occur more often with H3K9me2 and H3K9me3 (that is, H3K9me2S10ph and H3K9me3S10ph) than with H3K9un and H3K9me1, which we observed.

Our histone H4 characterization focused on lysines 5, 8, 12 and 16 acetylation and lysine 20 mono-, di- and tri-methylation (Figure 4). We observed that various cell lines, namely C33a, hESC2, HCT116 and U2OS, contained elevated levels of histone H4 acetylation, such as the tetra-acetylated H4K5acK8acK12acK16ac form and H4K5unK8unK12acK16ac. Enrichment for specific acetylated patterns may have interesting implications for Brd2, Brd3 and Brd4 recruitment, as discussed later. In contrast, another set of lines, namely the MCF7, MDA-MB231 and SW480, has depleted levels of histone H4 acetylation. It is interesting to recall that these same three cell lines also were enriched in H3K27 methylation and depleted in H3K9ac, H3K18ac and H3K23ac (Figure 3).

Finally, we briefly report our approximation of the distribution of H2A variants across the 24 lines (Additional file 3). There was little difference in abundance of macroH2A and H2AZ among most cell lines, although the trend suggests a greater abundance of H2A, in general, over macroH2A. For instance, H1229 (Wilcoxon rank sum, z = 2.6421 *P* = 0.0082) and hESC (Wilcoxon rank sum, z = 2.8022, *P* = 0.0051) possessed significantly more H2AZ than macroH2A. A notable exception is MDM13, which has significantly higher levels of macroH2A relative to H2AZ (Wilcoxon rank sum, z = −2.1651, *P* = 0.0304).

### Integration of both genomic and proteomic chromatin classifications of cell lines

In general, the genomic classification of the 24 lines was consistent with the proteomic classification of the 24 lines. Lines with similar expression profiles would generally have similar histone PTM patterns, and conversely, lines with distinct expression profiles would generally have distinct histone PTM patterns. Interestingly, the shortest-link clusters in the microarray data correlate well with the shortest-link clusters in the histone H3 PTM data. Specifically, the clusters (1) HEK293 and U2OS, (2) HFF, HeLa and PC3, and (3) MCF7, PANC1 and MDA-MD231 from the microarray data (Figure 1) reappear as clusters in the histone H3 PTM data (Figure 3). This consistency suggests that in these cell lines the mRNA levels of the histone modifying enzymes correlates well with their levels of enzymatic activity.

**Figure 5:**
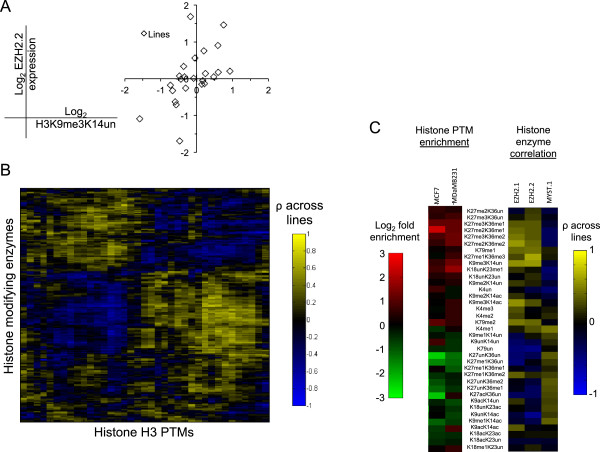
**Cross-correlation between the genomic and proteomic data. (A)** Example of cross-correlation analysis between the gene expression of a given enzyme (in this example, EZH2.2 variant) to the histone PTM log_2_ abundance (in this example, H3K9me3K14un) across all 24 lines (diamond). **(B)** Hierarchical clustering of Pearson correlation coefficients for each enzyme and histone H3 PTM cross-correlation analysis. For instance, a positive correlation would indicate that increased enzyme transcript level is associated with increased PTM level. **(C)** Comparison between enrichment of histone H3 PTMs for MCF7 and MdaMB231 (same data as found in Figure 3) and the correlation between those same H3 PTMs with EZH2 (EZH2.1 and EZH2.2) and MYST1 expression. Note, for instance, the enrichment of a given PTM (for instance, K27me3K36me2) in the two lines is generally accompanied by a positive correlation of EZH2, and a negative correlation of MYST1, for that PTM.

What one can learn uniquely from both the transcriptomic and proteomic experiments is to cross-correlate the expression levels of every histone modifying enzyme with the enrichment of every histone PTM across all 24 lines (Figure 5). The aim of such an analysis is to identify histone PTMs positively or negatively co-regulated by a particular enzyme. Not only should such cross-correlation procedures map particular modifications for a given enzyme with the histone residues it is known to modify, but also this approach should reveal novel modification sites targeted by a given enzyme *in vivo*. We further analyzed the expression profiles by bi-clustering [17] within individual cell line-clusters in Figure 1 to extract correlative behavior across different cell lines. For instance, re-clustering the expression profiles over only HEK293 and U2OS (the shortest link cluster in Figure 1) reveals that MYST1 and CDY1, both H4 HATs, are among the highest expressed transcripts for these two cell lines (Additional file 4). This finding is consistent with the observed increase in H4 acetylation in the U2OS (Figure 4).

A very similar result is obtained when bi-clustering over only HCT116 and SW480 (two colon cancer cell lines clustered together in Figure 1), where over an eight-fold increase in the H4 HATs CDY1 and HAT1 is observed (Additional file 5), and notably HCT116 is found to be enriched in H4 acetylation (Figure 4). Bi-clustering the expression profiles over the PC3, HFF and HeLa cell line-cluster reveals significant yet consistent changes in HMTs within these cells lines (Additional file 6). In particular, SETD6, PRDM5 and PRDM15 are the most significantly over-expressed (over 3.5-fold) and PRDM12, MLL2 and SUV420H1 are the most significantly under-expressed (over 3.5-fold) across all 224 transcripts. Several interesting patterns are also observed when bi-clustering the expression profiles within the breast cancer-rich cluster (that is, clustering only within the MCF7, PANC1, MDA-MB231 cell lines). Within these cell lines, a number of HMTs are found to be consistently and significantly up-regulated (Additional file 7). Namely, SUV420H1 (H4K20me), MLL2 (H3K4me), WHSC1 (H4K20me, H3K36me2), PRDM16 (H3K9me1), MECOM (H3K9me1), EZH2 (H3K27me2/3) and NSD1 (H3K36me) are all observed to exhibit higher expression levels in the MCF7, PANC1 and MDA-MB231 cell lines. The later pair of HKMTs, EZH2 and NSD1, are particularly interesting as these cell lines also exhibit the greatest increase in the hypermethylated states of H3K27K36 (that is, H3K27me2K36me2, H3K27me3K36me2, H3K27me2K36me1 and H3K27me3K36me1 are the most abundant in these cell lines in Figure 3). This correlative behavior between EZH2 and NSD1 potentially suggests a direct or indirect functional relationship between the two methyltransferases. Conversely, within the same cell lines there is a substantial decrease in the HATs GTF3C4 (H3K14ac), MYST1 (H4K16ac) and CDY1 (H4ac), and this is consistent with the observed decrease in H4 acetylation in the MCF7 and MDA-MB231 breast cancer cell lines (Figure 4).

As highlighted in the bi-clustering analysis, one of the more striking observations is that the two breast cell lines in our collection, namely MCF7 and MDA-MB231, are equally well characterized by both histone PTMs and the expression level of the associated enzymes. In particular, histone marks that were relatively enriched in the two cell lines, such as H3K27me3K36me1 and H3K9me3K14un, were also positively correlated with EZH2 expression. Interestingly, EZH2 is also positively correlated with H3K9me3K14un and H3K9me3K14ac, which may reflect binding of polycomb repressive complex PRC1 (which does not contain EZH2 HMT) with H3K27me3 and H3K9me3, as previously documented. It has been suggested that SUV39H1, an H3K9 methyltransferase, plays a role in targeting the PCR1 complex to H3K27me3 [18]. A more recent study analyzing the function of EZH2 in breast and prostate cancer lines has reported that the recruitment of EZH2 and Suz12 (both PRC2 subunits) to the promoter region of the tumor-suppressor gene *RKIP* is accompanied by H3K27 and H3K9 trimethylation [19]. These two studies support our observed correlation between the methylation levels on H3K9 and H3K27, particularly within the context of breast and prostate cancer.

As a reference, nearly the opposite correlations were observed for MYST1, a HAT reported to acetylate H4K16. MYST1 is negatively correlated with the aforementioned marks yet positively correlated with H3K27acK36un and H3K9acK14un. Indeed, this correlation makes sense from what is currently known regarding the relationship between histone PTMs and active gene transcription, as H4K16ac is implicated in transcriptional elongation [20], and H3K27ac and H3K9ac are marks that associate with active regulatory regions [21]. Taken together, our data point to the possibility that mammary cancer cells share a chromatin state characterized by elevated H3K27me3 levels and EZH2 expression. This suggested to us that EZH2 would be a particularly attractive candidate to investigate with respect to these two breast cancer cell lines.

### H3K27 methylation levels affect tumor formation in a mouse xenograft model

**Figure 6:**
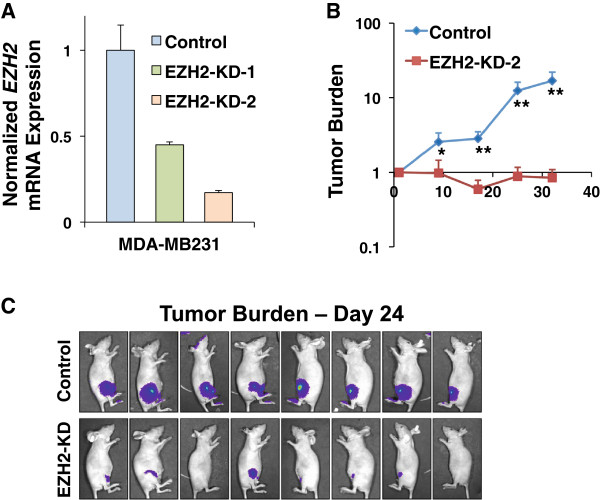
**EZH2 promotes breast cancer tumorigenesis *in vivo. *(A)** EZH2 was stably knocked down via shRNA in MDA-MB-231 cells. Knockdown efficiency was assessed via quantitative RT-PCR. **(B)** Control or EZH2-KD MDA-MB-231 cells were xenografted into mammary fat pads of nude mice and tumor burden was quantified over time via weekly bioluminescent imagine (BLI). BLI signals are normalized to the Day 1 signal for each mouse. **(C)** Images of mice injected with control or EZH2-KD cells are displayed (24 days post-injection). Data are shown as the mean + SD (RT-PCR) or mean + SEM (xenografts). *P <0.05; **P <0.01.

One of the most salient results from the proteomic atlas of chromatin modifications is enrichment of H3K27me3 in the MCF7 and MDA-MB231 breast lines. The functional roles of H3K27me3 and EZH2 in cancer progression are incompletely understood, as EZH2 has been proposed to both promote and oppose cancer formation and progression. From our profiling of 24 different cell lines and from the observed influence of H3K27me status on overall clustering, we hypothesized that lines most enriched for H3K27me3 would be especially reliant on this modification for their tumorigenicity. To test this hypothesis and validate our approach experimentally, we performed *in vivo* mouse xenograft tumorigenesis assays. Here, we stably knocked down *EZH2* in MDA-MB231 cells and injected control or KD cells into the mammary fat pads of nude mice. Assessing tumor burden via non-invasive, quantitative bioluminescent imaging, we observed a striking loss of tumorigenicity in *EZH2*-KD compared to control cells (Figure 6). While breast tumors formed by control cells grew rapidly, reflecting the aggressive nature of MDA-MB231 cells, *EZH2*-KD cells either formed modest tumors with reduced growth rates or failed to initiate tumors altogether. Overall, these results confirm the role of EZH2 in promoting breast cancer tumorigenesis. More specifically, the magnitude of the observed phenotype suggests that cell lines, such as MDA-MB231, which harbors strongly elevated H3K27me3 levels, absolutely require high EZH2 activity for their ability to initiate and sustain malignancies.

## Discussion

In this report, we provide a proteomic atlas of cancer lines based on histone modification patterns, and a companion genomic atlas of the same lines based on expression of histone modifying enzymes. The primary advantages of microarray procedures, in the context of this and similar experiments, are minimal requirement for sample cell amount and unsurpassed high-throughput quantification of enzyme transcripts. The primary advantage of MS procedures is the ability to extrapolate the catalytic activity of those enzymes by quantifying at the protein level the abundances of the histone modifications themselves that are the products and substrates for the enzymes. Although not as sensitive as antibody-based characterization of histone modifications, MS procedures offer a highly quantitative, unambiguous and generally unbiased identification of histone modifications that often cannot be easily controlled for otherwise [22,23]. Other studies comparing cancer types have similarly taken advantage of proteomic approaches to characterize chromatin modification patterns [24-26].

Our findings are generally consistent with other reports that interrogated specific PTMs in certain cancers (Table 2). For instance, various cancers, including HL60, HCT116 and primary non-tissue culture cancers, have been reported to have low levels of H4K16 acetylation and H4K20me3 with respect to normal tissue [26]. Although our analysis does not provide an immediate comparison to normal tissue, we observed a general depletion of H4K20me3, although notably not in H4K16 acetylation in both HL60 and HCT116 (Figure 4). As another example, non-small cell lung cancers have been reported to have low levels of H3K9ac and H4K16ac [27]. Likewise in this study, one of our lung lines H1229 had reduced levels of H4K16ac independent of the acetylation states of H4K5, K8 or K12, and reduced levels of both H3K9acK14un and H3K9acK14ac. Yet, there is a considerable level of inconsistency among reports on histone modification levels, for instance, whether low levels of H3K18ac are linked to negative [28] or positive [29] prognosis. Much of the variability could be due to heterogeneity of cancer cells from patients or differences in methods [30].

**Table 2:** Comparison between several observed histone PTM levels in this study and other studies (Chervona and Costa 2012)

**Histone modification**	**Observation in this study**	**Observation in other studies**
H3K4me3	Elevated in HCT116 colon line	Elevated SMYD3 HMT levels in colorectal carcinomas [31]
H3K9me3	Reduced in NB4 and HL60 leukemia lines	Reduced in promoter regions of acute myeloid leukemia patients [32]
H3K9me3 and H3K9me3K14ac	Elevated in MDA-MB231 and MCF7 breast lines	Elevated in circulating nucleosomes of breast cancer patients [33]
H3K18acK23un	Reduced in 293 kidney and H1229 lung lines	Reduced in poor prognosis kidney and lung cancer patients [34]
H3K27me3	Elevated in SAOS bone line	Elevated EZH2 HMT expression in osteosarcomas [35]
H3K27me3	Elevated in MDA-MB231 and MCF7 breast lines	Elevated EZH2 HMT expression in breast cancer [19]
H3K36me1 and H3K36me2	Elevated in HL60 leukemia line	Elevated recruitment of NSD1 HMT translocation in acute myeloid leukemias [36]
H4K16ac	Reduced in MDA-MB231 and MCF7 breast lines	Reduced in patient breast tumors [28]
H4K20me2	Reduced in PC3 prostate line	Reduced in metastatic and castration-resistant prostate cancer [37]
H4K20me3	Reduced in H1229 lung line	Reduced in lung carcinoma progression [38]

Our results are in slight contradiction with what was observed in an immunohistochemistry study of the global Ezh2 and H3K27me3 levels in breast cancer tumors and cell lines [39], where Mda-MB231 (claudin-low subtype, ER-negative) was reported to have moderate expression of both EZH2 and H3K27me3, but MCF-7 (luminal subtype, ER-positive) was reported to have low expression of both EZH2 and H3K27me3. The differences between our results and this study could potentially be due to differences in the methodology used. Nonetheless, our findings support the observed overexpression of Ezh2 in breast and prostate cancer [19], as DUT145, a prostatic adenocarcinoma, was observed to exhibit significant overexpression of Ezh2.2 (Figure 1) and belongs to the cluster of tissues with elevated H3K27me3 levels (Figure 3).Our ability to specify combinatorial modifications using mass spectrometry provides a dimension of the data that is nearly intractable from previous reports yet is an important consideration especially for the design of pharmacological inhibitors. Various inhibitors targeting histone PTM binding proteins have been reported to have therapeutic effect towards specific cancers, for instance, the JQ1 molecule inhibiting Brd4 binding to histone H4 [9]. Another example illustrating the relevance of combinatorial histone modifications is the estrogen receptor co-activator TRIM24, which is overexpressed in breast cancer and associates with the specific combinatorial pattern H3K4unK23ac [40]. The methods utilized in this report could be of considerable importance in designing new small molecules to compete *in vivo* with specific histone modification patterns and in assaying how inhibition of a specific histone PTM binding or modifying protein would impact other histone modification abundances on a genomic scale.

Recently, there have been several fatty acyl PTMs discovered on histones, including lysine crotonylation, and other marks, such as lysine succinylation or malonylation [13,41]. These types of modifications have been observed by our mass spectrometry methodology, but they are extremely low level. We estimate that these marks, such as crotonylation, are found in most cell types as low as 0.01% in abundance (data not shown). In fact, the manuscripts first describing these marks on histones have used either extensive fractionation methods or pan-lysine antibodies to first enrich for these species before mass spectrometry detection. As they are low level, we find these marks very difficult to robustly quantify from run to run, and have omitted them from the current studies.

A complementary experiment and limitation in the current work is to map where in the genome each specific histone PTM is localized for each line via ChIP-seq and to cross-reference those genes with interesting histone modification patterns to their transcriptional states. Additionally, future investigations should apply the methods presented in this paper to cancer cells not utilized in tissue culture, but rather collected from patients or harvested across cancer development from primary to metastatic tumors. An interesting application would be in the further subclassification of tumors from the same lineage. Recent studies have shown the utility of gene expression data alone in defining new molecular subtypes in breast cancer tumors [42]. Finally, it is important to emphasize that the chromatin modification landscape is but one facet underlying the complex heterogeneity of cancer. As cancer is not a single disease, it is unlikely for cancer to be explained using a single theoretical framework. An accurate classification must consider other aspects, such as metabolism and global DNA methylation patterns. For example, decreased levels in certain microRNAs, such as miR101 and miR214, result in the overexpression of EZH2 in cancer [43,44], and thus factors governing mRNA stability and translation should also be measured.

## Conclusion

In this report, we applied comprehensive transcriptomic and proteomic analyses with an emphasis on chromatin modification patterns to classify 24 commonly used cell lines, the majority of which are cancerous. Our results support the hypothesis that chromatin states, as defined by the expression levels of both histone PTMs and the associated enzymes, can serve as a basis for the characterization of different cancer types. By systematically and unambiguously quantifying the major histone H3 and H4 modifications, one can determine which specific histone modification exhibits the most interesting trends, whether in terms of relative enrichment or in terms of co-variation across similar lines. Bi-clustering analysis showed an overall correlation between the abundance of many histone PTMs and the expression of relevant enzymes, illustrating the complimentary nature of proteomic and transcriptomic data. The techniques and results of this report should be readily extended to non-tissue culture and clinical contexts. Finally, while we recognize that not all molecular processes underlying cancer are necessarily related to chromatin-related effects, we believe that the heterogeneity of the histone code itself in terms of its chemical structures and associated functions should offer a uniquely informative vantage into cancer biology.

## Methods

### Cell culture

The 24 cell lines used in this study are described in Table 1. All lines were cultured at 37°C in 5% CO_2_, with media supplemented with 1% penicillin/streptomycin, 1% glutamax and 10% fetal bovine serum. For harvesting, suspension cells were centrifuged at 200 g at room temperature, washed with 1× PBS, and flash frozen in liquid nitrogen for long-term storage at −80°C. Adherent cells were incubated with 10× trypsin + EDTA for ≤5 minutes when the digestion was quenched with serum-containing media, washed with 1× phosphate buffered saline, and flash frozen using liquid nitrogen for long-term storage at −80°C.

### Histone protein extraction and preparation for MS analysis

All tissue culture lines were similarly processed for histone extraction [45], and the resulting histones were processed for mass spectrometric analysis as described previously [6].

### MS analysis/data analysis

Histone peptides were loaded via autosampler onto a fused silica capillary for reversed phase high performance liquid chromatography as described previously [6], and electrosprayed into a hybrid linear quadrupole ion trap-orbitrap (ThermoFisher Scientific, Carlsbad, CA, USA ) as previously described. For each line, at least three replicates were performed. Mass spectrometric files for the lines can be accessed (Additional file 8) on the TRANCHE database (https://proteomecommons.org/tranche/). Statistical analyses were performed using MATLAB (R2012a). The LC-MS/MS data sets were analyzed using software that has previously been described elsewhere [17,46]. Briefly, an optimization-based model considers the MS isotopic distribution, MS/MS fragment ions and relative peptide hydrophobicity relationships to simultaneously identify and quantify (based on integrated peak areas) all modified forms of the same histone peptide. All charge states for modified peptides were included in the analysis, and the abundance of isobaric co-eluting modified forms were deconvoluted based upon tandem MS data as previously described [46]. All reported identifications were validated by manual inspection of the LC-MS/MS data.

### In-house designed microarray

Agilent’s eArray (Agilent Technologies, Inc., Cedar Creek, TX, USA) was employed to design oligo probes and assemble a 22 K-feature microarray. A customized set of 224 histone modifying genes (HAT, HDAC, HMT and HDM) and 551 house-keeping genes were selected to supplement a background consisting of the Agilent Human 1A gene set (Additional file 9). Four different probes were selected for each of the customized gene sets using vendor-supplied probe design processes and associated algorithms. A full list of the probes and features are available for download (Additional file 9).

### mRNA extraction and preparation for microarray analysis

Total RNA was isolated from all cell pellets by the RNeasy RNA isolation kit (Qiagen Sciences, Germantown, MD, USA, ). Equal amounts of RNA isolated from two duplicate wells were combined before the complimentary RNA (cRNA) amplification. The cRNA was then amplified using the Agilent low RNA input linear amplification kit (Agilent Technologies). The quantity and quality of cRNA were evaluated by capillary electrophoresis using an Agilent Technologies 2100 Bioanalyzer. Probe labeling and microarray hybridizations were performed as described in the Agilent 60-mer oligo microarray processing protocol (Agilent Technologies). Cy3-labeled probes derived from each of the 24 cell lines were co-hybridized against Cy5-labeled probes derived from a common reference RNA sample on the in-house designed oligonucleotide array described above. The common reference RNA consisted of a pooling of RNA isolated from all 24 cell lines; thus ensuring that the common reference probes were produced in the same fashion as the other probes, apart from the incorporation of different fluorescent dyes. Slides were scanned with an Agilent microarray scanner and image analysis performed using Agilent Feature Extractor Software. GeneSpring (Agilent Technologies, Inc., Santa Clara, CA, USA) v.9.0 was used to extract mRNA expression data for a total of 4,893 human genes, including the custom gene set; these mRNA expression data were used for correlation and functional analyses downstream.

### Tumor xenograft

All animal work was performed in accordance with the guidelines of the Institutional Animal Care and Use Committee of Princeton University under approved protocols. For tumor formation assays, MDA-MB231 cells were harvested from tissue culture and resuspended at a concentration of 1 × 10^7^ cells/ml in PBS. An incision was made in the abdomen of female athymic Ncr-nu/nu mice and 1 × 10^5^ cells (10 μl) were injected into the #4 mammary fat pad of each mouse. Noninvasive bioluminescent imaging (BLI) was performed weekly using an IVIS 200 Imagine System (Caliper Life Sciences, Alameda, CA USA) to quantify tumor burden over time. BLI data analysis was performed with Living Image Software (Xenogen, Alameda, CA USA) by measuring photon flux in the region of interest. Data were normalized to the signal obtained immediately after injection (Day 1).

### Reverse transcription and quantitative PCR

Total RNA was isolated using the RNeasy kit (Qiagen Sciences) and reverse-transcribed with the Superscript III kit (Invitrogen Life Technologies Co., Carlsbad, CA USA) following the manufacturer’s instructions. Quantitative PCR was performed in triplicate using the SYBR Green PCR Master Mix (Applied Biosystems, Warrington, UK) with the ABI Prism 7900HT thermocycler (Applied Biosystems). Raw *EZH2* expression levels were normalized to *GAPDH* expression levels. The following primers were used for qPCR: human EZH2 forward (5′-GTGGAGCCGCTGACCATTGGG-3′) and reverse (5′-CCTGCCACGTCAGATGGTGCC-3′), and human GAPDH forward (5′- GAAGGTGAAGGTCGGAGTC-3′) and reverse (5′- GAAGATGGTGATGGGATTTC-3′).

## Abbreviations

ac: Lysine acetylation; BLI: Bioluminescent imaging; cRNA: Complementary RNA; EZH2: Enhancer of zeste 2; HAT: Histone acetyltransferase; HDAC: Histone deacetylase; HDM: Histone demethylase; HMT: Histone methyltransferase; LC-MS/MS: Liquid chromatography tandem mass spectrometry; me1: Lysine monomethylation; me2: Lysine dimethylation; me3: Lysine trimethylation; PcG: Polycomb-group; PTM: Post-translational modification.

## Competing interests

The authors declare they have no competing interests.

## Authors’ contributions

GL, PM, BZ, IF and EC performed mass spectrometry experiments. GL, EC and BZ performed microarray experiments. MAB performed mouse experiments. GL, PD, BZ, MAB, EC, YK, PT and BG participated in the conceived experiments, created the study design and performed statistical analyses. GL, BZ, PD and BG drafted the manuscript. All authors read and approved the final manuscript.

## Supplementary Material

Additional file 1 **Relative abundance of histone H3 and H4 modified peptides across all cell lines and replicates.** These additional files are renumbered due to the shift in position of the previous Additional files 1 and 2 in the Methods section to the end of the manuscript. Click here for file

Additional file 2 Relative abundance of H3S10 phosphorylation across the cell lines, normalized across all the 20 H3 9 to 17 peptides. Click here for file

Additional file 3 Relative abundance of canonical H2A, H2AZ, macroH2A and H2AX quantified across all the cell lines, approximated by normalizing the different peptides unique to each protein directly to each other. Click here for file

Additional file 4 Up- and down-regulated enzymes in the HEK293 (neuronal precursor) and U2OS (bone) cell lines. Click here for file

Additional file 5 Up- and down-regulated enzymes in the HCT116 (colon) and SW480 (colon) cell lines. Click here for file

Additional file 6 Up- and down-regulated enzymes in the PC3 (prostate), HFF and HeLa (cervical) cell lines. Click here for file

Additional file 7 Up- and down-regulated enzymes in the MCF7 (breast), PANC1 (pancreatic) and MDa-MB231 (breast) cell lines. Click here for file

Additional file 8 **Mass spectrometric files for the 24 tissue culture cell lines can be accessed with the four hash tags on the TRANCHE database (**https://proteomecommons.org/tranche/**).** Click here for file

Additional file 9 List of genes probed in custom microarray and their expression in this study, with an overrepresentation of known human histone modifying enzymes among other housekeeping genes for all 24 tissues culture cell lines. Click here for file
