# Supplementary material for: A quantitative atlas of histone modification signatures from human cancer cells
Source: Epigenetics Chromatin. 2013 Jul 5;6:20. doi: 10.1186/1756-8935-6-20 (PMC3710262; doi:10.1186/1756-8935-6-20)
Supplement: Additional file 2 — Relative abundance of H3S10 phosphorylation across the cell lines, normalized across all the 20 H3 9 to 17 peptides. [file 1756-8935-6-20-S2.pdf]

	293	A549	C33a	DUT145	H1229	HaCAT	HCT116	HELA	HESC	HFF	HL60	Huh7	Huh75	MCF7	MDA-MB231	MDM	NB4	PANC	PC3	SAOS	SW480	U2OS	U251	U937
K9unS10unK14un	11.0	11.2	17.5	6.9	14.6	10.0	7.9	14.5	9.7	13.3	11.0	29.3	19.1	15.9	8.4	17.3	37.1	10.0	14.1	8.6	8.6	22.3	12.4	19.7
K9me1S10unK14un	8.5	9.8	16.0	8.0	11.3	9.1	6.3	12.3	9.2	13.5	9.0	32.2	50.3	8.2	7.4	14.4	3.2	8.3	24.0	9.7	6.3	16.6	10.5	10.3
K9me2S10unK14un	18.1	25.2	9.8	24.2	24.4	26.6	19.5	32.3	21.3	26.7	21.8	7.2	7.5	23.8	25.6	30.4	18.3	26.0	31.8	32.4	22.6	13.1	20.6	24.0
K9me3S10unK14un	10.8	20.1	8.2	32.6	21.8	16.2	16.2	18.7	21.0	20.4	11.5	11.2	8.7	20.0	31.3	14.4	6.2	23.6	10.6	19.9	33.8	9.4	11.0	11.2
K9acS10unK14un	1.4	0.2	2.6	0.3	1.0	0.3	1.0	0.8	1.5	1.3	2.4	1.9	1.1	0.7	0.3	1.0	1.0	0.8	2.3	1.4	0.6	1.0	1.1	0.8
K9unS10unK14ac	12.8	7.3	14.2	2.6	7.0	6.8	8.8	3.9	9.4	5.1	7.3	9.3	5.4	7.8	3.9	6.5	19.2	4.7	1.4	3.7	2.6	12.8	11.2	13.0
K9me1S10unK14ac	9.8	3.9	14.6	3.5	5.7	7.3	9.3	4.3	6.1	6.3	2.8	0.0	ND	4.0	3.9	5.1	4.7	1.7	3.1	2.8	3.0	13.0	9.4	6.4
K9me2S10unK14ac	18.8	14.4	8.4	9.0	8.6	18.3	18.1	9.1	14.9	9.7	23.1	4.2	3.1	13.3	9.3	8.8	6.1	12.4	4.4	13.6	10.3	4.5	16.7	10.6
K9me3S10unK14ac	6.8	6.2	6.6	12.4	4.5	4.0	11.1	3.1	5.4	3.2	9.9	1.0	0.5	5.4	8.9	2.3	3.6	7.7	8.1	6.5	11.9	5.9	4.8	3.3
K9acS10unK14ac	1.5	1.0	1.6	0.3	0.6	0.5	1.5	0.2	1.3	0.3	1.0	ND	0.1	0.4	0.4	ND	0.5	0.4	0.1	0.6	0.2	0.8	1.4	0.6
K9unS10phK14un	0.0	ND	ND	ND	ND	ND	ND	ND	ND	ND	ND	0.0	0.3	ND	ND	ND	ND	0.2	ND	ND	ND	0.0	ND	ND
K9me1S10phK14un	0.0	0.0	0.1	0.0	0.0	0.1	0.0	0.0	0.1	0.0	0.0	1.2	1.7	0.0	0.0	ND	ND	0.2	0.0	0.0	0.0	0.1	0.0	ND
K9me2S10phK14un	0.1	0.1	0.1	0.1	0.1	0.2	0.1	0.4	ND	0.1	0.1	0.5	1.9	0.0	0.0	ND	0.0	2.1	0.1	0.3	0.0	0.1	0.1	ND
K9me3S10phK14un	0.2	0.2	0.1	0.2	0.5	0.4	0.1	0.5	0.0	0.2	ND	0.2	0.2	0.1	0.3	ND	0.0	1.6	0.1	0.3	0.1	0.1	0.1	0.0
K9acS10phK14un	ND	0.4	0.0	ND	ND	0.0	ND	0.0	ND	0.0	ND	0.1	ND	0.0	0.1	ND	0.0	0.0	ND	0.0	ND	0.0	0.0	ND
K9unS10phK14ac	ND	ND	0.0	ND	ND	ND	ND	0.0	ND	0.0	ND	ND	ND	0.0	ND	ND	ND	0.0	ND	0.0	ND	0.0	ND	ND
K9me1S10phK14ac	0.0	0.0	0.2	ND	0.0	0.1	ND	ND	0.1	0.0	0.0	0.2	0.0	0.2	ND	ND	ND	0.0	ND	ND	ND	0.0	0.0	0.0
K9me2S10phK14ac	0.0	ND	0.0	0.0	ND	0.1	0.0	0.0	ND	0.1	0.1	ND	ND	0.0	0.0	ND	ND	0.3	0.0	0.1	ND	0.0	0.6	ND
K9me3S10phK14ac	0.1	ND	ND	0.0	ND	0.1	0.0	ND	ND	ND	0.1	ND	ND	0.0	0.0	ND	ND	0.1	ND	0.0	0.0	ND	ND	ND
K9acS10phK14ac	0.1	ND	0.0	0.0	0.0	0.0	0.0	0.0	ND	0.1	ND	1.5	0.1	0.1	0.0	ND	0.0	0.0	0.0	0.0	0.1	0.2	0.0	ND

*Notes: All values are normalized percentages
