# Supplementary figures and images for: A quantitative atlas of histone modification signatures from human cancer cells
Source: Epigenetics Chromatin. 2013 Jul 5;6:20. doi: 10.1186/1756-8935-6-20 (PMC3710262; doi:10.1186/1756-8935-6-20)

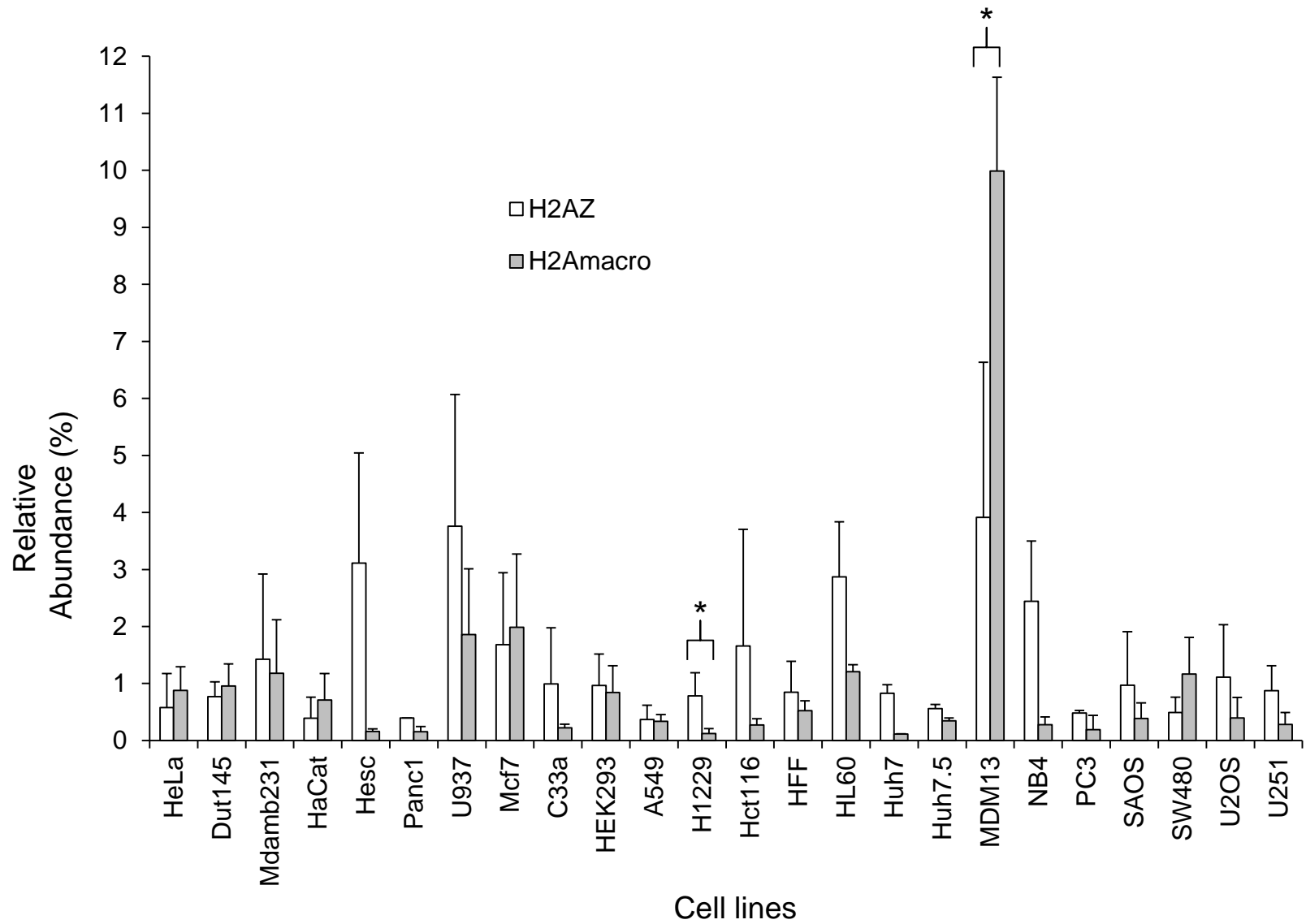

Supplement: Additional file 3 — Relative abundance of canonical H2A, H2AZ, macroH2A and H2AX quantified across all the cell lines, approximated by normalizing the different peptides unique to each protein directly to each other. [file 1756-8935-6-20-S3.pdf]

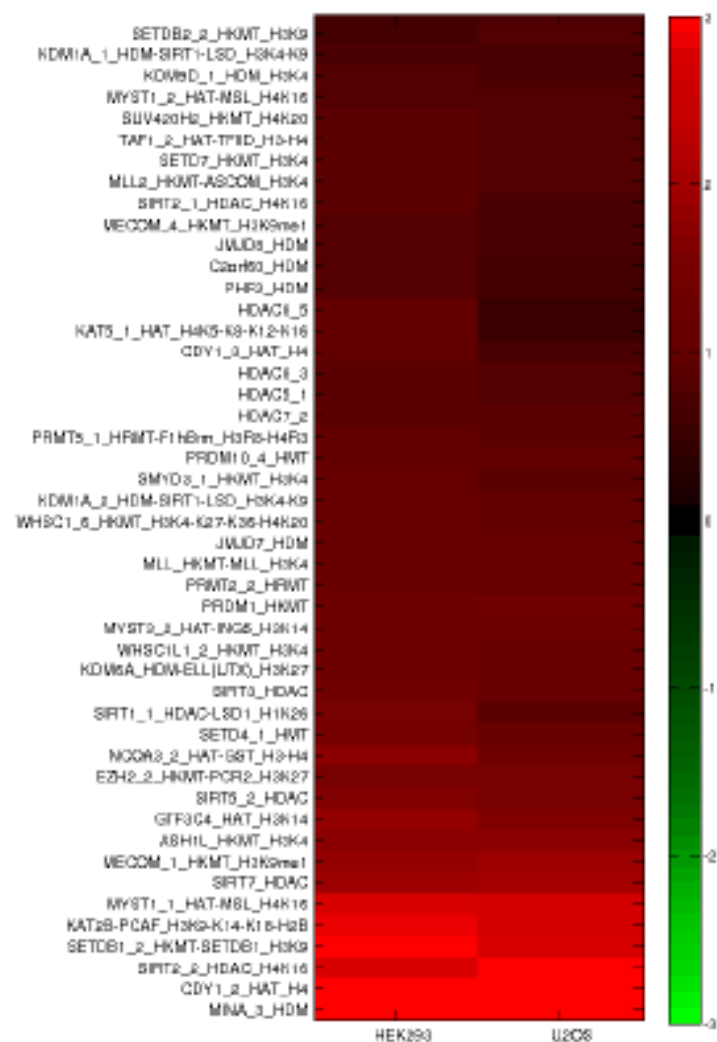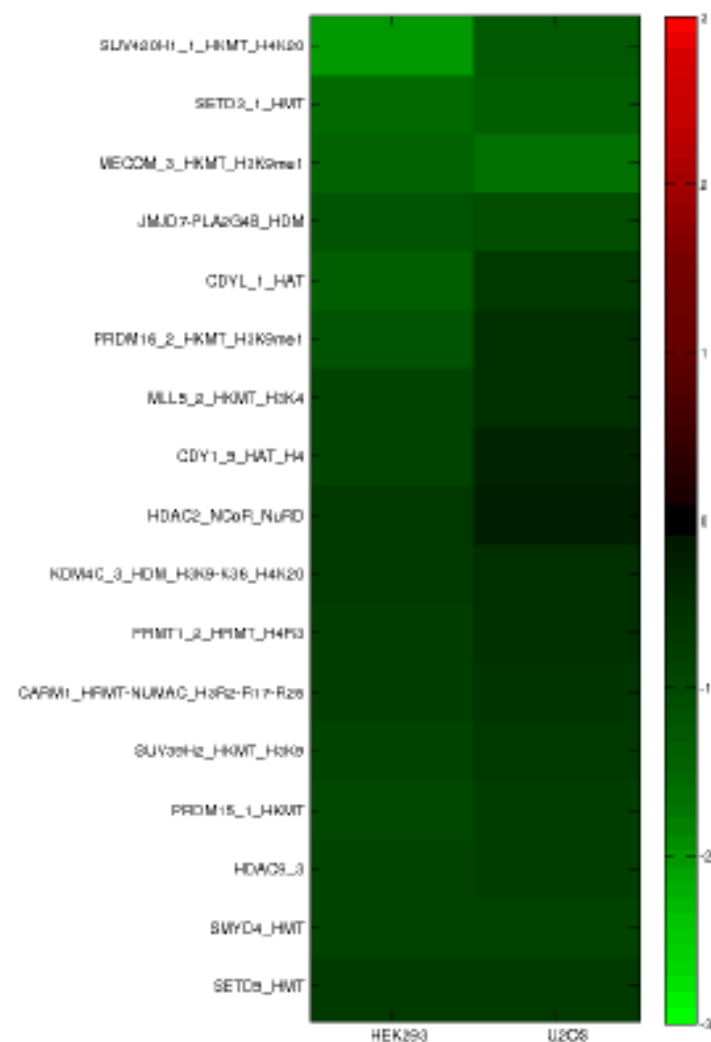

Supplement: Additional file 4 — Up- and down-regulated enzymes in the HEK293 (neuronal precursor) and U2OS (bone) cell lines. [file 1756-8935-6-20-S4.pdf]

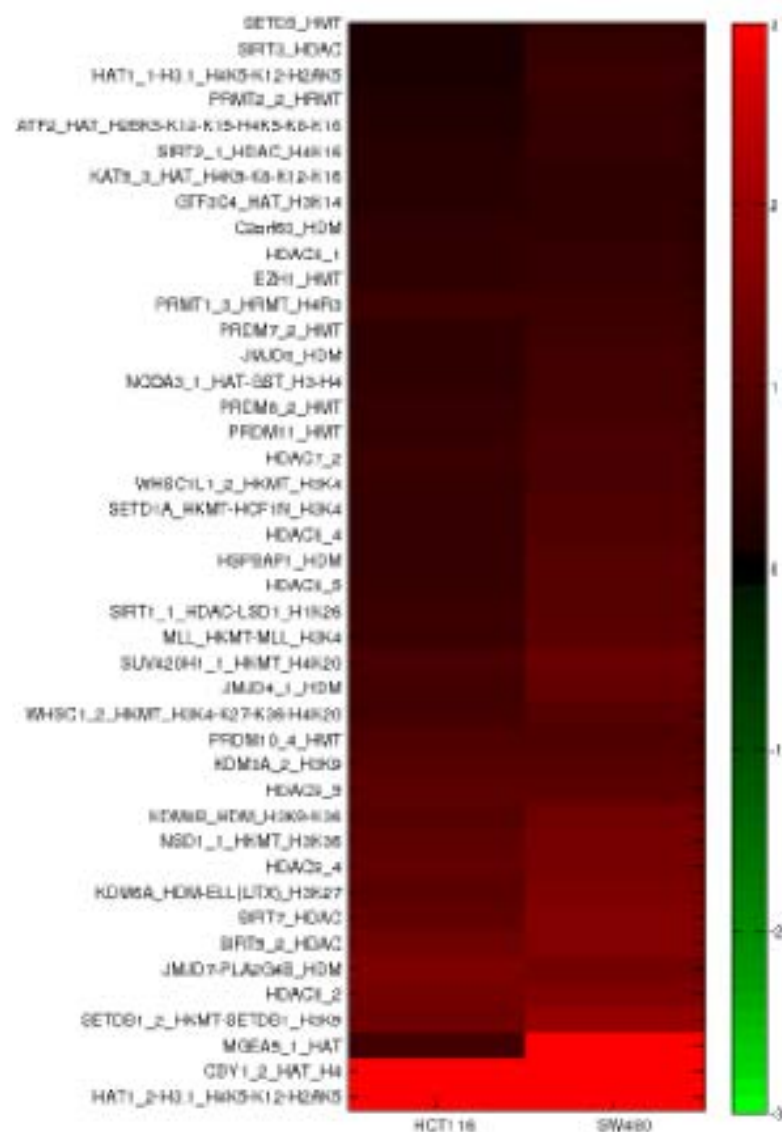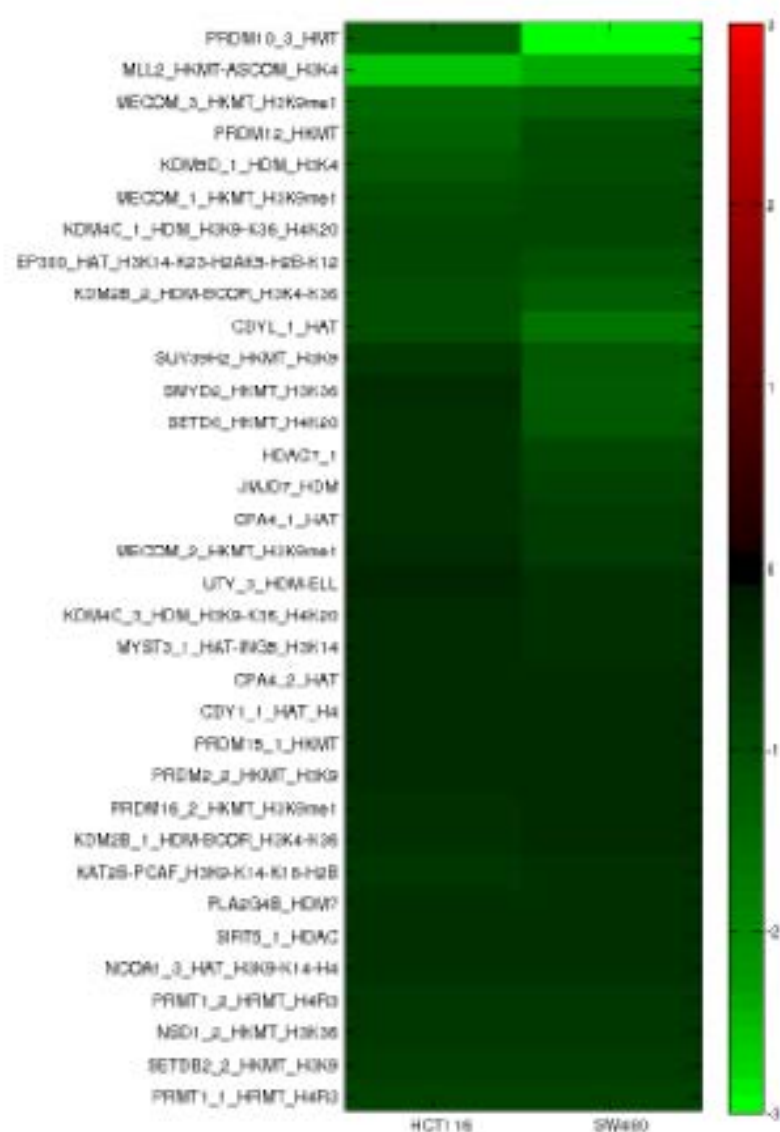

Supplement: Additional file 5 — Up- and down-regulated enzymes in the HCT116 (colon) and SW480 (colon) cell lines. [file 1756-8935-6-20-S5.pdf]

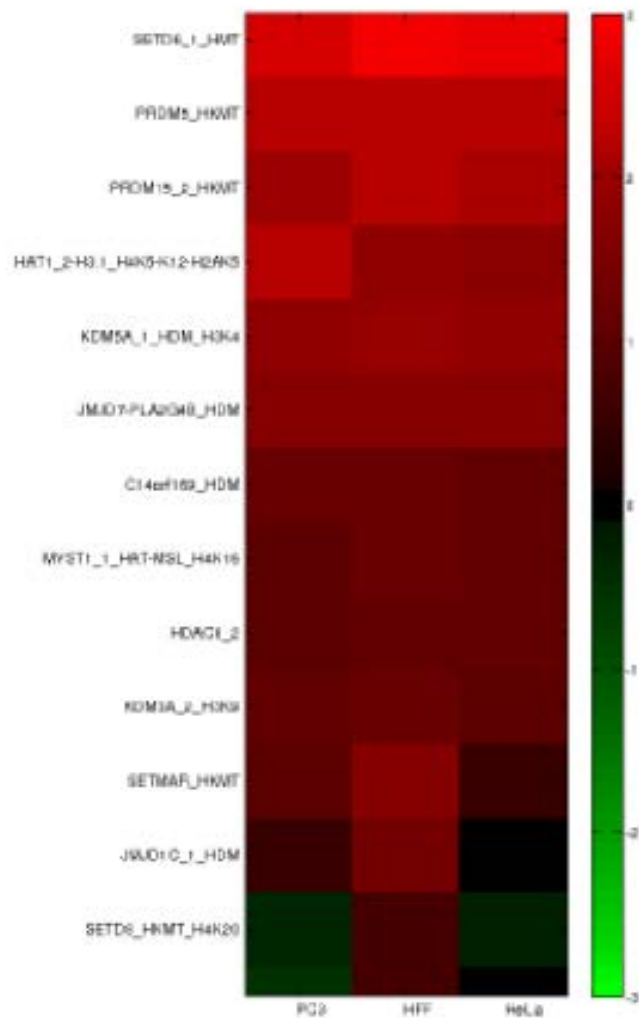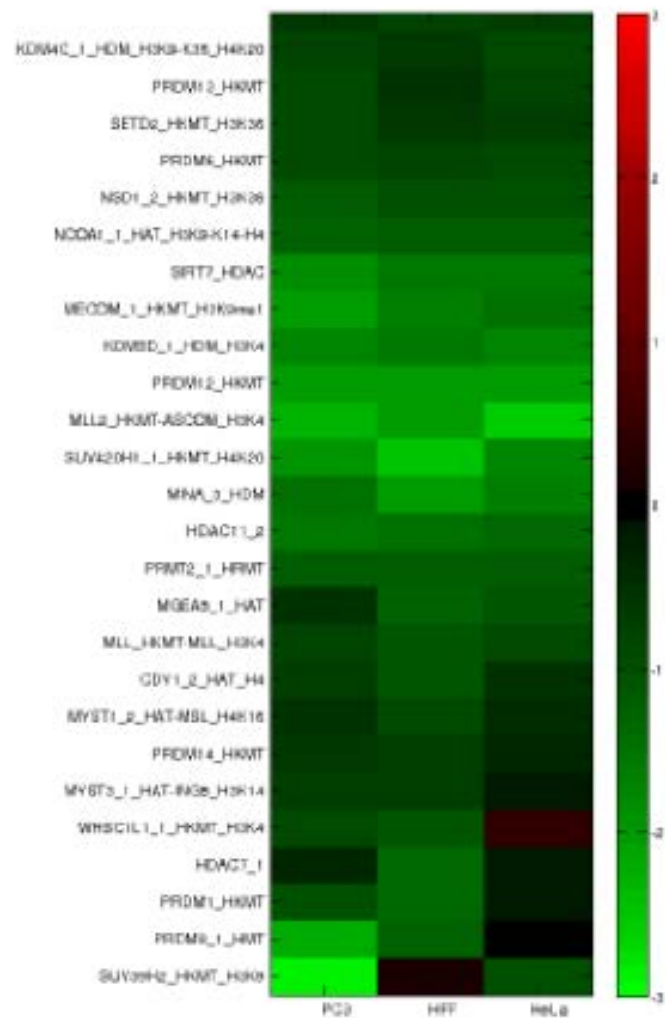

Supplement: Additional file 6 — Up- and down-regulated enzymes in the PC3 (prostate), HFF and HeLa (cervical) cell lines. [file 1756-8935-6-20-S6.pdf]

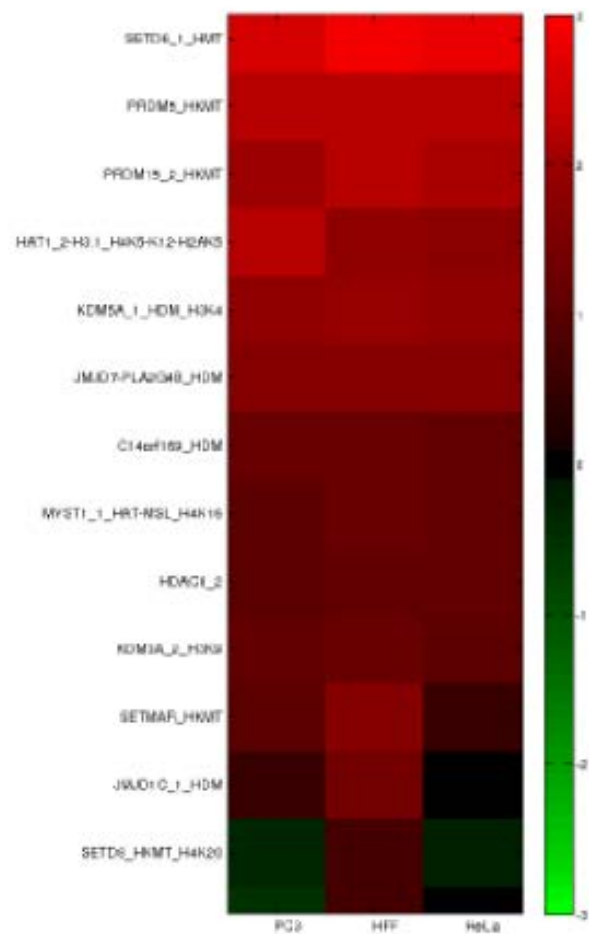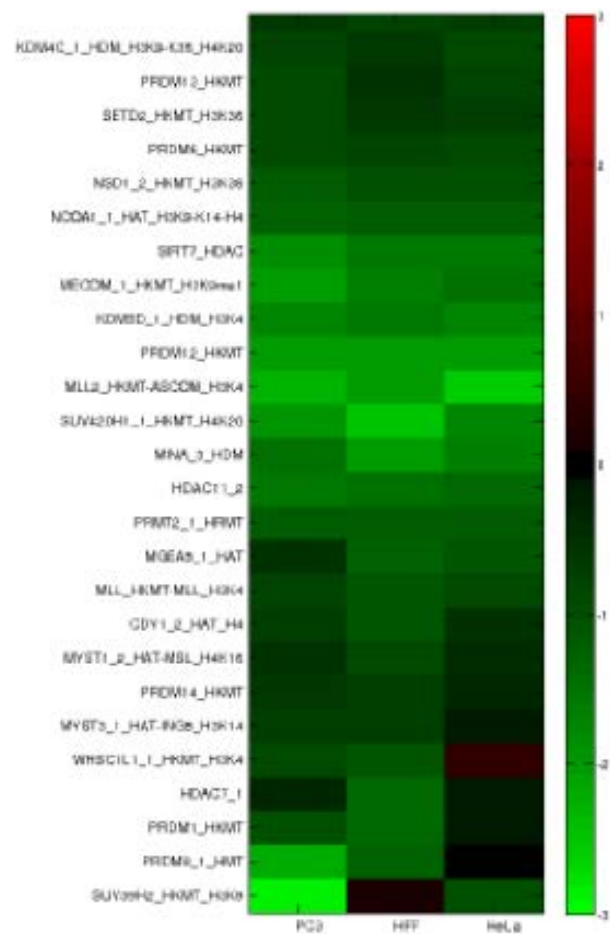

Supplement: Additional file 7 — Up- and down-regulated enzymes in the MCF7 (breast), PANC1 (pancreatic) and MDa-MB231 (breast) cell lines. [file 1756-8935-6-20-S7.pdf]
