# Supplementary material for: A quantitative atlas of histone modification signatures from human cancer cells
Source: Epigenetics Chromatin. 2013 Jul 5;6:20. doi: 10.1186/1756-8935-6-20 (PMC3710262; doi:10.1186/1756-8935-6-20)
Supplement: Additional file 8 — Mass spectrometric files for the 24 tissue culture cell lines can be accessed with the four hash tags on the TRANCHE database (https://proteomecommons.org/tranche/). [file 1756-8935-6-20-S8.pdf]

Additional File 1. Mass spectrometric files for the 24 tissue culture cell lines can be accessed with the four hash tags on the TRANCHE database (https://proteomecommons.org/tranche/).

1) wJ01iPpE16MCjsCtuJmLyccGdnCRxFc393jbbuIJLX1v6TFqqYyo0/vnDDJLrKvIosXVHjtfNO6/v0Neb7/TpCHfi0IAAAAAAAABcQ==

2) Sb30XrB62O9oZf1t2P/ZfPdlahd/AI5JRmv5sEUZDfDyxmRk+56gIIQNx8VdDDL+44IXnGdVJ7hVQLYOZl7kObkxdwwAAAAAAAABpw==

3) ijbCk/hM2dWsY5awdh89LY/RFffvvaKeu+T0izui7tqFfNFgqjGsGb5z3d1a+eeaTqctBadd2ikHKK09+tCeRyoCiYUAAAAAAAABpQ==

4) /NjXrj34gQVIbzxp3UIUXycxtn35y/uQleXmLuQJdnoIyAXlgoZN2TXAJi+dALgjKRyGOhxCIDSr7pbXgRNFgjuIAQoAAAAAAAABqw==
